# Microbial-Based Plant Biostimulants

**DOI:** 10.3390/microorganisms11030686

**Published:** 2023-03-07

**Authors:** Mohamed Hijri

**Affiliations:** 1 Département de Sciences Biologiques, Institut de Recherche en Biologie Végétale (IRBV), Université de Montréal, Montreal, QC H1X 2B2, Canada; mohamed.hijri@umontreal.ca; 2 African Genome Center, Mohammed VI Polytechnic University (UM6P), Ben Guerir 43150, Morocco

Beneficial microorganisms offer essential ecological services to both natural and agricultural ecosystems [1,2]. As their potential for improving agricultural productivity is immense [2], utilizing these microbes is a major aim of environmental and agricultural biotechnologies. Achieving this could help us promote more efficient soil nutrient usage; thereby, increasing crop yield and quality while minimizing the environmental footprint of agriculture. In the past few years, the world has made great strides in developing novel technologies to ensure food production while mitigating the greenhouse gas emissions of agriculture. Plant biostimulants, which are defined here as microbial-based inoculants, substances derived from organisms (e.g., microbes, plants of animals), or a combination of both, used to improve nutrient uptake, protect plants from biotic and abiotic stress, and promote growth (e.g., germination, flowering, pollinator recognition and attraction, fructification, maturity, and crop quality), have become increasingly popular. Although these biostimulants have been widely utilized in farming, horticulture, and forestry, several scientific issues remain unresolved. This Special Issue on Microbial-Based Plant Biostimulants seeks to address these questions and explore the effectiveness of microbial-based plant biostimulants and their potential influence on the indigenous microbial communities of soils and the plant microbiota (Figure 1).

A study by Mahdi et al. (2022) [3] has been carried out on a halotolerant phosphate solubilizing bacterium associated with quinoa plants. The bacterium, *Bacillus velezensi* QA2, was charactrrized through metabolic screening and it was found to solubilize both inorganic phosphate (P) and zinc, produce indole-3-acetic acid, ammonia, hydrogen cyanide, and cellulase, as well as to form biofilms. It was also shown to withstand high salt concentrations (11% NaCl). When tested on quinoa plants grown in saline conditions, *B. velezensi* QA2 was found to promote growth and reduce the effects of saline irrigation. Chlorophyll index and P content significantly increased in inoculated plants, while sodium concentrations decreased significantly. The authors also performed a bibliometric analysis that revealed the current scope of research on *B. velezensis* strains. The overall findings indicate potential for the use of *B. velezensis* as a biostimulant to improve plant growth, control pathogen attacks, and reduce the salinity impact on quinoa crops [3].

Another study by Goncalves et al. (2022) [4] used an oligotrophic medium to isolate potential novel soil bacteria that interact positively with soybeans. Twenty-two species of bacteria from the soybean rhizosphere were chosen, consisting of both known genera and potentially novel species. These bacteria showed plant growth-promoting (PGP) traits both in vitro and improved soybean growth under drought in a greenhouse experiment. The draft genome sequences of the *Kosakonia* sp. strain SOY2 and *Agrobacterium* sp. strain SOY23 were also reported. Analysis of comparative genomics of 169 genomes belonging to the genera reported by Goncalves et al. (2022) [4] indicated that the bacteria possess genes encoding PGP proteins and gene clusters for secondary metabolite production, which directly affect plant growth. These findings make it possible to identify novel soil bacteria, opening the door for their use as microbial-based plant biostimulants.

Complex interactions between beneficial microorganisms, crops, herbivores, and their natural predators and parasitoids are important factors of trophic cascades and have a major impact on community diversity and functioning in agroecosystems (Figure 1). Two different studies investigated these complex interactions using soybean (*Glycine max* L.) as a model crop. The first investigation [5] showed that the co-inoculation of the arbuscular mycorrhizal fungus *Rhizophagus irregularis*, and the nitrogen (N)-fixing bacterium *Bradyrhizobium japonicum* on soybean, positively influenced the performance of *Aphis glycines*. The authors observed that the double inoculation increased the plant’s biomass, nodulation, mycorrhizal colonization, N and C concentrations, but decreased the P concentration. In addition, the *B. japonicum* inoculation alone had similar effects, except for root biomass. Only an increase in mycorrhizal colonization and P concentration was observed with the inoculation of *R. irregularis* alone. A heightened reproductive rate was seen in the aphids with the double inoculation and the *B. japonicum* alone, with no effect noted with *R. irregularis*. The size of individual aphids was not changed. They also noted a positive correlation between N concentration and aphid population density. The findings of the study of *Dabre* et al. (2022) [5] demonstrated that the combination of both symbionts can have a greater effect on the performance of both the plant and the insect than when each are introduced alone. The second study explored the effect of these inoculatants (*R. irregularis* and *B. japonicum*), in soybean on two natural enemies of *Aphis glycines* [6]. They recorded the growth and survival of the predator, *Coleomegilla maculata*, and the parasitoid *Aphelinus certus*, reared on aphids feeding on soybean inoculated seedlings. Results showed that the inoculation with *B. japonicum* alone decreased the rate of parasitoid emergence, likely due to reduced host quality, yet the number of mummies, sex-ratio, development time, and parasitoid size remained unchanged. Inoculation with *R. irregularis* alone or when co-inoculated with *B. japonicum* had no influence on any of the parameters of the parasitoid. Similarly, none of the parameters of the predator were impacted by any inoculant. The findings suggest that the benefits of the microbe–plant symbioses to the second trophic level hardly extend to the third trophic level [6].

It is difficult to predict the effect of introducing Arbuscular mycorrhizal fungi (AMF) inoculants in the field on indigenous mycorrhizal communities. Based on analysis of 15 studies, a review of the literature suggests that AMF inoculants can persist in the amended field for a few months to several years, though usually with an abundance that declines over time, or complete exclusion [7]. It also appears that AMF inoculation can have both positive and negative impacts on native AMF species, including suppression, stimulation, exclusion, and neutral impacts. Various factors, such as the inherent properties of the inoculum, dosage and frequency of inoculation, and soil physical and biological factors, influence the success of AMF inoculants. To ensure the success of commercial inoculants and reduce discrepancies among studies, it is important to improve and standardize sampling methods and the molecular tools used to quantify AMF taxa. Furthermore, inoculant producers should focus on selecting strains with a higher chance of success in the field and little or no downstream impacts.

Interventions with commercial inoculants are intended to reduce the environmental impact of agricultural chemical inputs, but their applications have sparked worries about unintended microbial invasions. As there are no reports on AMF invasion, Basiru and Hijri (2022) [8] assessed the framework used to define it and provide insight into how to prevent negative impacts. Although commercial AMF isolates can be considered potential invaders, invasions may not always lead to reduced native community diversity and functions. The success of the invaders and the present communities is determined by ecological processes such as selection, drift, dispersal and speciation. To lower reliance on introduced inoculants, they suggest implementing management practices that boost diversity and abundance of native AMF, and using native propagules as a supplement to commercial AMF in suitable locations. Additionally, controls need to be put in place to monitor inoculant quality and composition, as well as their movement between distant regions.

Lastly, the availability of P is a major limiting factor in crop production. To improve soil P fertility, plants can rely on their microbiomes, especially on microbial P-solubilizing inoculants that have been suggested as a way to increase P availability in agroecosystems. To further research this interaction between the soil–plant–microorganism interface and the P cycle, a comprehensive review of the literature was carried by Ducousso-Detrez et al. (2022) [9]. On one hand, the diversity of P-chemical forms in soils was studied, as well as the various factors that shape and drive these forms. On the other hand, an analysis was conducted to understand how P affects microbial community diversity in soils. Even though a direct link between P bioavailability and microbial community composition has yet to be established, P has been identified as a major factor in the presence/absence and/or abundance of certain bacterial taxa. The authors concluded that a better understanding of the relationship between soil P chemistry and soil microbiology is needed to effectively utilize microbial-based inoculants in sustainable agriculture.

## Figures and Tables

**Figure 1:**
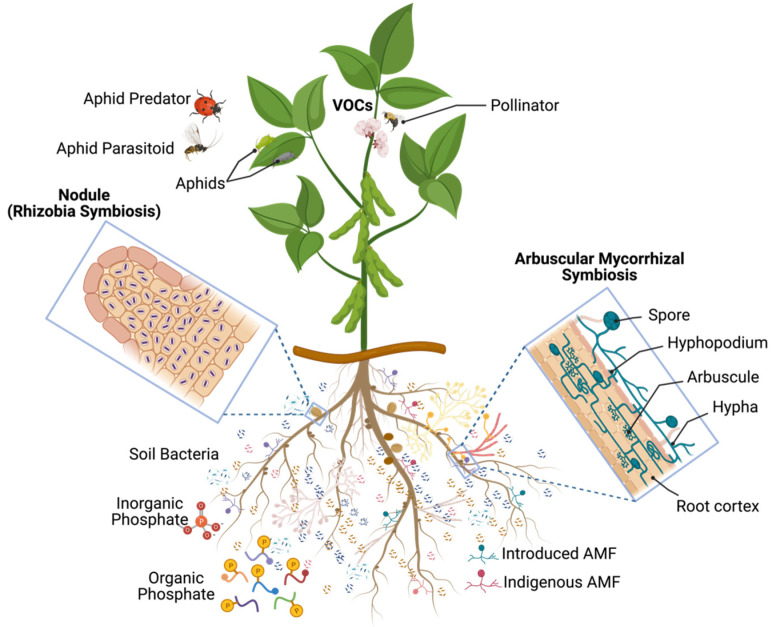
Overview of studies on microbial-based plant biostimulants and trophic cascades of interactions on soybean used as model crop. VOCs, volatile organic compounds; AMF, arbuscular mycorrhizal fungi (Created with BioRender.com).
